# The Arabic version of the hospital survey on patient safety culture: a psychometric evaluation in a Palestinian sample

**DOI:** 10.1186/1472-6963-13-193

**Published:** 2013-05-24

**Authors:** Shahenaz Najjar, Motasem Hamdan, Elfi Baillien, Arthur Vleugels, Martin Euwema, Walter Sermeus, Luk Bruyneel, Kris Vanhaecht

**Affiliations:** 1 Health Services Research Group, School of Public Health KU Leuven, Leuven, Belgium; 2 Faculty of public health, Al-Quds University, Jerusalem, Palestine; 3 Human Relations Research Group, HU Brussel, Brussels, Belgium; 4 Research Group Work, Organizational and Personnel Psychology KU Leuven, Leuven, Belgium

## Abstract

**Background:**

A growing global interest in patient safety culture has increased the development of validated instruments to asses this phenomenon. The aim of this study is to investigate the psychometric properties of the Hospital Survey on Patient Safety Culture (HSOPSC) and its appropriateness for Arab hospitals.

**Methods:**

The 7-step guideline of the Agency for Healthcare Research and Quality was used to translate and validate the HSOPSC. A panel of experts evaluated the face and content validity indexing of the Arabic version. Data were collected from 13 Palestinian hospitals including 2022 healthcare professionals who had direct or indirect interaction with patients, hospital supervisors, managers and administrators. Descriptive statistics and psychometric evaluation (a split-half validation technique) were then used to test and strengthen the validity and reliability of the instrument.

**Results:**

With respect to face and content validity, the CVI analysis showed excellent results for the Arab context (CVI = 0.96). As to construct validity, the 12 original dimensions could not be applied to the Palestinian data. Furthermore, three of the 12 original dimensions were not reliable (*α* <0.6). The split-half technique resulted in an optimal 11-factor model.

**Conclusions:**

Our study is the first study in the Arab world to provide an evaluation of the HSOPSC using Arabic data from Palestine. The Arabic translation of the HSOPSC comprises an 11-factor structure showing good validity and acceptable reliability. Despite the similarity between the Arab factor structure of the HSOPSC and that of the original one, and taking into account that our version may be applied in Arabic hospitals, there is a need for caution in comparing HSOPSC data between countries.

## Background

Patient safety is a global public health topic. WHO estimates that millions of patients worldwide suffer from disabling injuries or death due to unsafe medical care [1]. To date, limited data on unsafe medical care in the Arab world is available. Wilson et al. assessed the frequency and nature of adverse events in Egypt, Jordan, Kenya, Morocco, Tunisia, Sudan, South Africa and Yemen. Findings suggest that 8.2% of records reviewed showed at least one adverse event, ranging from 2.5% to 18.4% per country. Eighty-three percent of these adverse events were judged to be preventable (range 55%-93%). About 30% of adverse events were associated with death of the patient. This equates to nearly 2% of patients in hospital across the eight countries sustaining an adverse event that was associated with their death [2]. As a consequence, El-Jardali identified enhancing the quality of healthcare services as a research priority in the Middle East [3]. In similar vein, WHO and its partners launched a call for studies that may help to improve patient safety [4], for example by validating instruments that measure safety culture.

Among initiatives to advance patient safety, growing interest has been given to patient safety culture. As stated in the Institute of Medicine’s report ‘To err is human’, safety culture properly promotes - and thus enhances - patient safety [5]. The Joint Commission for Accreditation of Healthcare Organizations included an annual assessment of safety culture in its 2007 patient safety goals [6]. Such assessment provides information on aspects of the organizational culture (the underlying values, beliefs and norms; e.g., the way we communicate around or work together in an organization) that underlie active failure in patient care and on latent conditions (e.g., unworkable procedures, poor or inadequate technology, understaffing) that should be addressed by patient safety initiatives [7-9].

Patient safety culture has been measured by a range of tools that evaluate dimensions such as communication, teamwork and attitudes to errors. For most of these instruments, however, evidence on validity and reliability properties is rather limited or even non-existent [6]. One of the most applied instruments is the Hospital Survey on Patient Safety Culture (HSOPSC), a tool developed by the Agency for Healthcare Research and Quality (AHRQ) [10]. It is widely translated and validated in a broad range of countries such as UK, The Netherlands, Belgium, Scotland and Norway [10-15]. Recently, also Arabic hospitals, particularly in Jordan, Sudan and Lebanon, have applied an Arabic translation of the HSOPSC [16]. So far, there is however no international published evidence regarding the validation of this tool for the Arab world; an issue we want to address in our current study.

The aim of this study is to validate the Arabic version of the HSOPSC and to provide Arab hospitals with a well-structured, consistent and psychometrically sound instrument to measure patient safety culture. To this aim, we investigated face validity, content validity, construct validity and reliability of the HSOPSC/Arabic Version (AV). The validated instrument should guarantee that the instrument assesses the important dimensions of patient safety culture also in Arabic hospitals. Such validated instrument would allow researchers to compare the safety culture across hospitals at the national and international level.

## Methods

**Figure 1:**
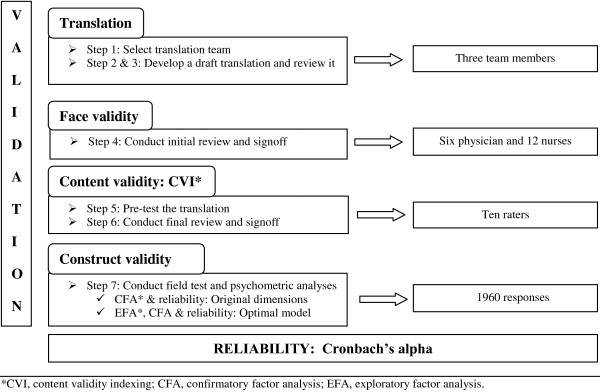
Overview of the validation and reliability analysis of the HSOPSC/AV.

The original HSOPSC consists of 42 items on 12 dimensions: two outcome dimensions and 10 safety dimensions. Respondents address these 42 items by means of a five-point Likert scale of which the labels vary throughout the dimensions; 1 = ‘strongly disagree’ to 5 = ‘strongly agree’, or, 1 = ‘never’ to 5 = ‘always’ [17]. Before starting any translation process, the available translated Arabic version of HSOPSC was implemented and revised in a pilot study. Some items were incomprehensible and others have translation issues in some items, like items *A5* (Staff in this unit work longer hours than is best for patient care) and *B4* (My supervisor/manager overlooks patient safety problems that happen over and over). Consequently, we decided to translate the original survey again by following AHRQ guidelines for translating survey on patient safety culture [18]. These guidelines propose a team approach based on current best practices for survey translations [18-22]. Moreover, they prepare for a validation and a reliability process, which we aimed to adopt in our study. In order to develop and validate an HSOPSC/AV, we followed the seven steps as described in the translation guidelines of AHRQ (Figure 1) [18]. Translation and validation methods are described in the following sections.

### Translation process

To develop a well-translated HSOPSC/AV, the original survey was translated by following steps 1-3 of the 7-step guidelines for the AHRQ survey on patient safety culture (Figure 1) [18]. Forward and back translation were performed as a standard part of translating surveys. To develop and review the Arabic translated draft, a translation process was followed by our translation team. This team consists of a bilingual translator with professional work experience in developing surveys, a bilingual reviewer with experience in translation and a translation coordinator.

### Face and content validity

After translating the survey into Arabic, we investigated the face and content validity of the HSOPSC/AV in steps 4-6 (Figure 1). To obtain *face validity*, six physicians and 12 nurses from two hospitals conducted an initial review and signoff for the Arabic translation of the HSOPSC. They met to review the translation, suggest changes and decide about the most suitable translation. Next, an expert panel of ten raters - nine from five hospitals and the quality coordinator from the Palestinian Ministry of Health - was recruited to obtain *content validity* indexing (CVI). They determined whether the questions from the pre-final Arabic version suited the Arab culture and if the format of the questions was conceptually equivalent to the original English question [23]. First, they assessed the cultural relevance of the questions using a four-point Likert scale (not relevant = ‘1’ to highly relevant = ‘4’). Second, they assessed the quality of the translation using a binary “Yes/No” question that evaluated whether the items were semantically and technically equivalent. Based on these aspects, CVI-scores were calculated: one for the cultural relevance of the scale and one for the semantic and technical equivalence of the scale.

### Sample and its properties

The construct validity of the final Arabic translation was tested by means of a survey between September 2010 to August 2011 in 13 Palestinian hospitals; all general public hospitals and two general non-governmental hospitals in West Bank. The Hospital sampling of non-governmental hospitals was based on comparable hospitals that serve large communities and have, the same general departments. The survey targeted all healthcare professionals who had direct or indirect interaction with patients, all hospital supervisors, managers and administrators. A paper version of the questionnaire was distributed via participants’ mail boxes. Participants were informed about the purpose of the study and their participation was anonymous, voluntarily and confidential. Moreover, one point of contact was appointed in each hospital, so that the hospital’s staff had one central source of assistance in case they had questions or concerns about the survey. The project was fully supported by the Palestinian Ministry of Health (MoH), the hospital administration and the quality departments within the hospitals. We obtained the approval of the ethics committee and permits from MoH (The Palestinian ministry of healthcare: Healthcare research committee) and hospitals to carry out this assessment. Incentives were not provided to participants for completing the survey.

The survey was returned by 2022 participants (response rate = 53.6%); 62 respondents did not fill out all the questions and were therefore omitted from the study. Finally, 1960 questionnaires were retained for further analysis. Most of the respondents (88.9%) had direct interaction or contact with patients. 50% of the sample had worked more than six years in their current hospital. Most respondents were nursing staff (51.3%), followed by physicians (17.7%), management and administrative staff (10.5%), technicians (9.4%), related healthcare professionals (5.4%), and finally other (4.7%). These percentages give a reasonable reflection of the real distribution of disciplines in each of the departments.

### Statistical analysis

In view of performing confirmatory factor analysis (CFA), exploratory factor analysis (EFA) and reliability scores; we revised the codes of negatively worded items so that a higher score always reflected a more positive response. In line with other validation studies on HSOPSC [8,11,13], CFA were used to investigate whether the factor structure of the original 12 dimensions of patient safety culture could be applied to our set of data collected in Arabic health care setting. The purpose was to confirm that the existing scales/dimensions may be reasonably used within the Arab context. CFA was used to assess the overall level of fit for the whole data sampling. The fit indices that were used for CFA were; Comparative Fit Index (CFI >0.90 acceptable or >0.95 good fit), Tucker-Lewis index (TLI >0.90 acceptable or >0.95 good fit), Root Mean Square Error of Approximation (RMSEA < 0.08 acceptable, ≤ 0.05 good model of fit) and the Standardized Root Square Residual (SRMR < 0.08 good fit model) [24,25]. Since model fit proved unsatisfying, the data were subsequently analyzed with EFA to examine whether the items represent different factors in the Arabic data. For that purpose, the total sample was randomly split in two parts; the first part (sample I, n = 960) was used to inspect the factor model of the items and to provide alternative data-based scales using EFA, while the second part (sample II, n = 1000) was used to conduct CFA to assess how well our Arabic data can be modeled using existing scales, not by themselves alone but compared to scales determined directly from those data by EFA. Finally, we assessed the internal consistency of the adapted and optimal Arabic version for the entire dataset. The reliability of the factors was evaluated by means of Cronbach’s alpha (α). A reliability greater than or equal to 0.6 indicates that the items measure the same concept [17].

## Results

### Face and content validity

The pre-survey review from our face validity team led to a number of changes in the wording or structure of some items to improve understandability and readability. All original items were kept to be able to allow comparison of our results with other studies using the HSOPSC. The suggestions and comments of this team resulted in a pre-final Arabic version, which was then used to evaluate the content validity. The scale-CVI score yielded 0.80, indicating an acceptable cultural relevance [23]. The translation CVI was excellent and reached 0.96. Two items (A5, B4, see Table 1) however showed a problematic rating on the translation, and were presented to the raters for additional review. The final Arabic translation was then distributed in hospitals to assess the construct validity.

**Table 1:** **Factor loadings**, **standard path coefficient CFA and Cronbach**’**s alphas of the HSOPSC**/**AV**

**Factor/items and its Cronbach’s alpha**	**11 Factors**											**Standard path coefficient CFA**
	**1**	**2**	**3**	**4**	**5**	**6**	**7**	**8**	**9**	**10**	**11**	
**Factor 1: Teamwork within departments (α = 0.77)**												
A1: People support one another in this unit	0.81											0.73
A3: When a lot of work needs to be done quickly, we work together as a team to get the work done	0.77											0.77
A4: In this unit, people treat each other with respect	0.76											0.71
A11: When one area in this unit gets really busy, others help out	0.60											0.56
**Factor 2: Supervisor/manager expectations and actions promoting patient safety (α = 0.75)**												
B1: My supervisor/manager says a good word when he/she sees a job done according to established patient safety procedures		0.53										0.74
B2: My supervisor/manager seriously considers staff suggestions for improving patient safety		0.60										0.81
B3: Whenever pressure builds up, my supervisor/manager wants us to work faster, even if it means taking shortcuts		0.79										0.50
B4: My supervisor/manager overlooks patient safety problems that happen over and over		0.83										0.68
**Factor 3: Hospital hand-offs and transitions (α = 0.73)**												
F3: Things “fall between the cracks” when transferring patients from one unit to another			0.63									0.58
F5: Important patient care information is often lost during shift changes			0.77									0.71
F7: Problems often occur in the exchange of information across hospital units			0.76									0.63
F11: Shift changes are problematic for patients in this hospital			0.65									0.61
**Factor 4: Frequency of event reporting (α = 0.87)**												
D1: When a mistake is made, but is caught and corrected before affecting the patient, how often is this reported?				0.82								0.81
D2: When a mistake is made, but has no potential to harm the patient, how often is this reported?				0.86								0.87
D3: When a mistake is made that could harm the patient, but does not, how often is this reported?				0.82								0.80
**Factor 5: Feedback and communication openness about error (α = 0.73)**												
C2: Staff will freely speak up if they see something that may negatively affect patient care					0.74							0.66
C4: Staff feel free to question the decisions or actions of those with more authority					0.71							0.49
C3: We are informed about errors that happen in this unit					0.62							0.72
C5: In this unit, we discuss ways to prevent errors from happening again					0.50							0.69
**Factor 6: Staffing (α = 0.75)**												
A2: We have enough staff to handle the workload						0.78						0.80
A5: Staff in this unit work longer hours than is best for patient care						0.77						0.73
A14: We work in "crisis mode" trying to do too much, too quickly						0.79						0.65
**Factor 7: Organizational learning – continuous improvement (α = 0.80)**												
A6: We are actively doing things to improve patient safety							0.86					0.88
A9: Mistakes have led to positive changes here							0.87					0.87
A13: After we make changes to improve patient safety, we evaluate their effectiveness							0.63					0.56
**Factor 8: Overall perceptions of safety (α = 0.75)**												
A15: Patient safety is never sacrificed to get more work done								0.87				0.88
A18: Our procedures and systems are good at preventing errors from happening								0.88				0.86
A17: We have patient safety problems in this unit								0.56				0.36
**Factor 9: Hospital management support for patient safety (α = 0.66)**												
F8: The actions of hospital management show that patient safety is a top priority									0.65			0.70
F9: Hospital management seems interested in patient safety only after an adverse event happens									0.57			0.36
F1: Hospital management provides a work climate that promotes patient safety									0.69			0.76
**Factor 10: Teamwork across hospital departments (α = 0.61)**												
F4: There is good cooperation among hospital units that need to work together										0.76		0.61
F10: Hospital units work well together to provide the best care for patients										0.77		0.62
F2: Hospital units do not coordinate well with each other										0.43		0.45
F6: It is often unpleasant to work with staff from other hospital units										0.61		0.47
**Factor 11: No punitive response to error (α = 0.60)**												
A16: Staff worry that mistakes they make are kept in their personnel file											0.67	0.60
A8: Staff feel like their mistakes are held against them											0.69	0.60
A12: When an event is reported, it feels like the person is being written up, not the problem											0.75	0.50

### Testing the original model (12-factors): CFA and reliability analysis

A CFA that applied the original HSOPSC dimensions to the Arabic translation (χ2 (753) = 2294, *p* < .001) revealed a rather satisfactory fit: CFI = 0.91, TLI = 0.90, RMSEA = 0.04 and SRMR = 0.05. Nevertheless, looking at the path coefficients, not all of the items loaded significantly on the assumed dimension of the HSOPCS. We therefore decided to carry out an EFA in order to determine whether there is an optimal model with factor structure that better fits the Arabic data.

### Testing the alternative model (11-factors): EFA versus CFA

We applied a cross-validation technique by randomly splitting the sample into two complementary subsets. The first subset, ‘the training subset’, was used to construct an optimal model for the HSOPSC/AV. The second subset, ‘the test subset’, was used to validate the model and to control for possible overfiting of the data. The exploratory factor analysis was conducted on the 42 items with Varimax rotation on the training subset. Kaiser-Meyer-Olkin (KMO) verified that the sampling adequacy for the analysis was high = 0.85 [26], indicating that there is hardly any spread in the correlation pattern, enabling reliable and distinctive dimensions by factor analysis. Additionally, all KMO values for individual items were above the acceptable limit of 0.5 [26]. Bartlett's test was highly significant (p < 0.001), which indicated that correlations between items were sufficiently large for EFA. An initial analysis was run to obtain Eigenvalues for each component in the data. Eleven dimensions had Eigenvalues over Kaiser´s criterion of 1 and in combination explained 61, 44% of the variance. Number of factors to be extracted was confirmed by Scree plot results. Table 1 shows the EFA’s factor loadings. Three items did not load on any of the factors; namely ‘It is just by chance that more serious mistakes don’t happen around here’ (A10), ‘We are given feedback about changes put into place based on event reports’ (C1), and ‘Staff are afraid to ask questions when something does not seem right’ (C6). Moreover, the reliability of ‘staffing’ increased from α = 0.66 to α = 0.75 when deleting the item ‘We use more agency/temporary staff than is best for patient care’ (A7). Furthermore, the items that previously formed “communication openness” and “feedback and communication about error” appeared as one dimension in the HSOPSC/AV. Based on these results, we optimized the model by (a) defining 11 instead of 12 dimensions, with one dimension being ‘communication openness and feedback about error’, and (b) omitting four items from the HSOPC/AV, namely A7, A10, C1 and C6.

The test subset was used then for a CFA in which we applied the optimal model to the HSOPSC/AV (χ2 (610) = 1375.518). This optimal model showed a good fit to the data; CFI = 0.91, TLI = 0.90, RMSEA = 0.04, and SRMR = 0.06. All path coefficients were significant (Table 1).

### Reliability

The internal consistency was calculated for the original facture structure. The reliability analysis of the 12 original dimensions is presented in Table 2. Of those 12 original dimensions, only one achieved α > 0.80 (Frequency of event reporting; α = 0.87). The other factors got an acceptable level of reliability (α ≥0.6, according to the AHRQ pilot study) [17]. Three dimensions achieved low reliability; namely, ‘No punitive response to error’ (α = 0.59), ‘communication openness’ (α = 0.41) and ‘overall perception of safety’ (α = 0.43). Table 2 also shows the reliability level of the Arabic translation version as compared to the original English HSOPSC. As a final test, the reliability level was examined for the new factor structure by using the whole dataset. The Cronbach´s alpha was satisfactory; 0.60 to 0.87 (Table 1). While eight factors showed a good reliability (more than 0.73), three factors had acceptable reliability (0.60 to 0.66) according to the AHRQ pilot study [17]. The overall Cronbach´s alpha coefficient was 0.87.

**Table 2:** Cronbach’s alphas of the HSOPSC/AV as compared to the HSOPSC

**Factor**	**No of items**	Cronbach’s alpha (α) HSOPSC *	**Cronbach’s alpha (α)** **HSOPSC/arabic version**
Teamwork across hospital departments	4	0.80	0.61
Teamwork within departments	4	0.83	0.77
Hospital hand-offs and transitions	4	0.80	0.73
Frequency of event reporting	3	0.84	0.87
No punitive response to error	3	0.79	0.59
Communication openness	3	0.72	0.41
Feedback & communication about error	3	0.78	0.69
Organizational learning – continuous improvement	3	0.76	0.79
Supervisor/manager expectations & actions promoting patient safety	4	0.75	0.75
Hospital management support for patient safety	3	0.83	0.66
Staffing	4	0.63	0.65
Overall perceptions of safety	4	0.74	0.43

*As published by AHRQ report [17].

## Discussion

Patient safety culture is an important determinant for patient safety in healthcare. To take into considerations cultural differences in measuring this concept and to allow national and international comparisons of research findings, we applied a widely used instrument to assess patient safety culture. This study is the first study in the Arab world which reports on the structure and psychometric properties of the HSOPSC/AV according to the guidelines of the AHRQ.

Although our results were in line with the original HSOPSC, we did introduce some small adaptations to really fit the Arab context. The main difference was that the original dimensions of “communication openness” and “feedback and communication about error” were grouped into one dimension. This result is reasonable because both dimensions are closely related. The fact that these items loaded on the same factor could underline that the Arabic wording of the items belonging to these two dimensions must be directed more towards the differences between communication openness and communication about error in future studies. In addition, we would recommend rewording the four items (A7, A10, C1, and C6) that were excluded from the CFA to derive more reliable scales.

With regard to limitations, a first limitation concerns the relatively low internal consistency of some scales compared to the original survey. This finding was already reported in earlier research from the Netherlands, Belgium, England, Scotland, Norway, and Turkey [11-15,27]. Another limitation could be that looking at different factor structures based on type of provider (multi-group factor analysis or factor analysis with covariates) has not been performed in our study. Such type of analysis can offer evidence whether factor structures are invariant across different types of providers. Although our factors in CFA were treated as correlated latent variables, our EFA using Varimax rotation treated factors as uncorrelated [14]. To examine the difference between the two techniques, additional EFA using oblique rotation was performed. This did however not change the factor structure. Therefore, our EFA results using Varimax rotation are in line and comparable to other American and European studies [14].

One of the strength of our study is that we applied the CVI technique. However, as far as we know, this technique has not always been used in other studies evaluating psychometric properties of HSOPSC. CVI ensures that the translated items match well the original items and determines if the questions were semantically and technically equivalent to the original English questions.

The correlation between patient safety culture and actual adverse events (patient harm) was not investigated in this study. Such a test would however be recommended, as it gives further insight in the criterion-related validity and may give indicate whether or not the tool prevents patient safety failures. In addition, it would further confirm the predictive validity of the instrument. Therefore, we are now in the process of collecting and analyzing patient safety data to conduct criterion-related validity for our survey.

Future research could extent this validation study by investigating the multilevel psychometric characteristics of the Arabic version in different Arab countries. Despite the fact that we used the standard Arabic language in our translation, which means that the version should be understood in all Arab countries, multilevel analysis approach will offer even more evidence. Another interesting research direction would be to include the understanding and the perception of healthcare leaders toward patient safety [28,29]. This could be done using methods as focus groups or interviews, due to the sensitive implications of such activity. Leadership support has high impact on promoting and adopting patient safety culture within hospitals.

## Conclusion

The results of our study revealed that HSOPSC/AV looks very similar to the original HSOPSC. Nevertheless, the factor structure was not identical and showed lower internal consistency comparing with the original HSOPSC. An optimal model becomes more acceptable and reliable by removing weak items and shifting others.

Our findings show the importance of a translation according to well-established steps. And that an instrument can only be correctly applied to measure safety culture when the different safety dimensions of the instrument are assed in a correct way, as validated within similar or comparable health systems either within one country or across countries [30,31]. This would encourage researchers to apply validated tools instead of non-validated translations, as the latter provide results on Patient Safety culture that cannot be compared with other national and international studies in this respect [13].

Based on the current findings, we may conclude that the HSOPSC/AV is a suitable instrument to assess the safety culture in the Arabic speaking hospital settings. Nevertheless, there is need for caution in benchmarking dimensional scores results between countries without taking in consideration the differences within the national and international healthcare settings and the psychometric evaluation of the translated versions.

## Abbreviations

HSOPSC: Hospital survey on patient safety culture; AHRQ: Agency for healthcare research and quality; CVI: Content validity indexing; WHO: World Health Organization; HSOPSC/AV: Arabic version; MoH: Palestinian ministry of health; CFA: Confirmatory factor analysis; EFA: Exploratory factor analysis; CFI: Comparative Fit Index; TLI: Tucker-Lewis index; RMSEA: Root mean square error of approximation; SRMR: Standardized root square residual; α: Cronbach’s alpha; KMO: Kaiser-Meyer-Olkin.

## Competing interests

The authors declare that they have no competing interests.

## Authors’ contributions

SN performed part of the statistical analyses of the data, coordinated the translation procedure of the questionnaire and drafted the manuscript. MH coordinated CVI, collected the data and revised critically the manuscript. EB performed part of the statistical analyses and contributed to the manuscript. AV and ME have participated in revising the manuscript critically for important intellectual content. WS and LB participated in the analyses and interpretation of the data, and contributed to the manuscript. KV was involved in the study design, manuscript outline and revised the manuscript critically. All authors read and approved the final manuscript.

## Pre-publication history

The pre-publication history for this paper can be accessed here:

http://www.biomedcentral.com/1472-6963/13/193/prepub
